# Effect of Chicken AvBD11 on the Cytokines in the Erythrocytes of Chickens Infected with the Avian Influenza Virus of the Subtype H9N2

**DOI:** 10.3390/ani15071023

**Published:** 2025-04-02

**Authors:** Jie Yu, Sheng-Qing Luo, Wen-Jun Xiang, Zi-Xuan Meng, Ying Wang, Jian-Le Ren, Yu-Jun Zhao, Rui-Wen Fan, Sheng Niu, Wen-Xia Tian

**Affiliations:** College of Veterinary Medicine, Shanxi Agricultural University, Jinzhong 030801, China; yuj126315@163.com (J.Y.); shengqingluo@163.com (S.-Q.L.); wangying@sxau.edu.cn (Y.W.); tgzhaoyujun@163.com (Y.-J.Z.); ruiwenfan@163.com (R.-W.F.)

**Keywords:** AvBD11, chicken erythrocytes, H9N2, cytokines

## Abstract

(1) The aim of this study was to elucidate the role of the *Gallus gallus* avian β-defensin 11 (AvBD11) in the immune response induced by the avian influenza virus H9N2. (2) AvBD11 was expressed using *E. coli*, and the effects of different concentrations of AvBD11 on cytokine expression in the ex vivo and in vivo erythrocytes of chickens infected with the avian influenza subtype H9N2 were detected by using fluorescence quantification. (3) The results showed that cytokine expression varied among the test groups compared to the control group in the in vitro assay at 2, 6, and 10 h. *Lipopolysaccharide induced TNF factor* (*LITAF*) and *Interferon-γ* (*IFN-γ*) were significantly increased in the AvBD11 group with the addition of the final concentration of 15 μg/mL at 6 h. At 10 h, *Interleukin-1β* (*IL-1β*) and *IFN-γ* were both more significantly increased in the 15 and 10 μg/mL groups than in the H9N2 group alone. In the in vivo test, *IFN-γ* and *Interleukin-10* (*IL-10*) were significantly increased in the high-dose group than in the H9N2 group at 3 d and 7 d. (4) In conclusion, the ability of AvBD11 to induce the expression of more cytokines by chicken erythrocytes in a short period of time suggests that it is not only an antimicrobial peptide but also a possible immunomodulator.

## Simple Summary

AvBD11 is an atypical, double-sized defensin predicted to have two motifs associated with β-defensins and six disulfide bonds. We expressed AvBD11 using *Escherichia coli* and examined the effects of different concentrations of AvBD11 on cytokine expression in ex vivo and in vivo erythrocytes from chickens infected with avian influenza subtype H9N2. The results showed that the addition of different concentrations of AvBD11 caused changes in cytokine expression at different times. It is suggested that AvBD11 is not only an antimicrobial peptide but may also be an immunomodulator.

## 1. Introduction

The H9N2 AIV was initially isolated from turkeys in Wisconsin in the United States of America in 1966 (A/turkey/Wisconsin/1/1966(H9N2) [1]. The H9N2 subtype of low-pathogenic avian influenza (LPAIV) is the most common LPAIV in poultry worldwide [2]. H9N2 viruses can cause harm to avian species through direct pathology, co-infection, and immunosuppression [3,4]. At the same time, the H9N2 virus not only directly infects humans but also contributes some or all of its internal genes to the emerging lethal variants of H5N1, H7N9, H10N8, and H5N6 [5], which pose a threat to public health. Therefore, research on the H9N2 virus deserves great attention.

Defensin proteins are small in size (less than 10 kDa), rich in cysteine (forming 3 to 6 disulfide bonds), and typically cationic (with a net charge interquartile range of +1 to +5) [6]. The defensins can be divided into the following three groups on the basis of their structure: α-, β-, and θ-defensins [7]. Avian β-defensins (AvBDs) are small, positive, non-glycosylated polypeptides with three lamellar structures attached to an α-helical loop [8,9], similar in structure to mammalian β-defensins [10]. *Gallus gallus* avian β-defensin 11 (AvBD11) is an atypical defensin found in birds, primarily expressed in the oviduct [11] and also present in eggshells and albumen. It constitutes one of the main components of the outer layer of the yolk membrane (VM), serving as the final protective barrier for the embryo [12].

Defensins have not only antibacterial but also antiviral properties. Human β-defensins have been reported to have direct inhibitory activity against a variety of viruses, including human immunodeficiency virus (HIV) [13] and influenza virus [14]. Co-incubation of pBD-3 with PRRSV significantly inhibited viral infectivity in MARC-145 cells. In the PAMs model, pBD-3 and PG-4 at concentrations of 5–40 μg/mL consistently suppressed PRRSV titers [15]. Cytokines are secreted or membrane-spanning molecules that mediate a wide range of cellular functions, including development, differentiation, growth, and survival. Therefore, the regulation of cytokine activity is important in both physiology and pathology [16]. In addition to carrying oxygen, erythrocytes participate in the body’s immune regulatory responses and can exert their immune functions through immuno-adhesion and other effects [17]. Human erythrocytes have been the focus of research in the field of erythrocyte immunity, while studies on the immune function of nuclear erythrocytes in animals have focused on fish and reptiles, but there are fewer studies on the involvement of chicken erythrocytes in the immune response of the organism. The HA protein of the highly pathogenic avian influenza virus (HPAI) is readily cleavable and exhibits robust hemagglutination activity. In contrast, H9N2, which falls under the LPAI category, possesses an HA protein with restricted cleavage capacity and a diminished ability to bind to red blood cells. Consequently, when compared to HPAI, there are notable differences in the mechanisms of red blood cell invasion and the subsequent immune responses elicited by the virus. The long-term epidemic of the H9N2 subtype AIV poses a significant threat to livestock, poultry production, and human health. This ongoing situation creates an environment that facilitates the emergence and spread of highly pathogenic or novel influenza strains. Based on the immune function of defensins, this experiment was proposed to investigate the changes of avian AvBD11 on the expression levels of various cytokines in H9N2-infected chicken erythrocytes, which will help in studying the antiviral function and immune mechanism of chicken defensin protein against H9N2 in more depth.

## 2. Materials and Methods

### 2.1. The Cloning of AvBD11

The pCold-AvBD11 plasmid was constructed using standard molecular biology techniques, with the pCold vector plasmid (TAKARA, Dalian, China) as the template. Total RNA from Hyland Brown egg yolks was extracted and reverse-transcribed for amplification of the AvBD11 coding sequence. Primers were designed using DNASTAR to amplify the mRNA coding region sequence of AvBD11, i.e., the mature sequence of AvBD11 (NM_001001779). The PCR product was amplified using the following specific primers: the forward primer was 5′-CATATGGAGCTCGGTACCCTCGAGTTACCGCGTGATACCAGCC-3′, and the reverse primer was 5′-CTGCAGGTCGACAAGCTTGAATTCTCAGATTTCTTTGCAGCAGGTCCAAC-3′. These primers contain restriction enzyme sites for both the *Xho*I and *EcoR*I enzymes (TAKARA, Dalian, China), which are underlined in the above sequences. The reaction was carried out using Phanta Max Super-Fidelity DNA Polymerase (Vazyme, Nanjing, China). The following conditions were used for PCR amplification: a pre-denaturation step at 95 °C for 3 min, a denaturation step at 95 °C for 15 s, an annealing step at 70 °C for 15 s, and an extension step at 72 °C for 35 cycles and finally 72 °C for 5 min. PCR products were recovered using the Omega Gel Extraction Kit and cloned into pCold vectors using the In-Fusion HD Cloning Kit (TAKARA, Dalian, China),and transformed *E. coli DH5α* (Sangon Biotech, Beijing, China) recipient cells according to the manufacturer’s instructions. The cells were cultured in Luria–Bertani (LB) solid medium (containing 100 μg/mL ampicillin) at 37 °C for a period of 12 h. Positive clones were isolated and cultured overnight in LB liquid medium (containing 100 μg/mL ampicillin). Plasmids were extracted using the Plasmid DNA Extraction Mini Kit (Tiangen, Beijing, China) and sequenced by Sangon Biotech (Shanghai, China). The successfully constructed plasmids were then transformed into *E. coli DE3* (Sangon Biotech, Beijing, China) and sequenced.

### 2.2. Production of Recombinant AvBD11 Protein

*E. coli DE3* was grown in LB liquid culture medium (containing 100 μg/mL ampicillin) at 220 r/min and 37 °C until the optical density (OD) reached 0.6 to 0.8. Subsequently, the recombinant protein expression was induced in bacterial cultures by the addition of 1 mM IPTG (Solarbio, Beijing, China) at 16 °C for 16 h. Following this, the bacterial cultures were subjected to centrifugation at 8000 r/min for 15 min. The bacteria were collected. Bacteria were resuspended in Tris-HCl (pH 8.0) and PMSF was added at a final concentration of 1 mM. Bacteria were fragmented using ultrasound and then centrifuged at 8000 r/min for 15 min to collect the upper layer of liquid, which was purified with His-labeled purification resin, and the target protein was eluted with 100 mM imidazole. Subsequently, the recombinant fusion protein AvBD11 was detected by Western blotting using His-Tag mouse monoclonal antibody (Huaxinbio, Beijing, China). The protein was then treated with HRV3C protease (TAKARA, Dalian, China), stirred at 4 °C overnight, and separated using Superdex™ 75 Increase (Cytiva, Uppsala, Sweden). Finally, SDS-PAGE was used for further analysis.

### 2.3. Cell Culture and Virus Strains

H9N2 viruses were obtained from our laboratory stock [18]. Specific pathogen-free (SPF) embryonated eggs used for virus propagation were purchased from Beijing Boehringer Ingelheim Vital Biotechnology Co., Ltd. (Beijing, China). The selected hemagglutination titer was 7 log_2_ (TCID_50_ = 10^4.63^/mL). All experiments in this study were approved by the Institutional Animal Care and Use Committee of Experimental Animal Management at the Shanxi Agricultural University, China (Approval No SXAU-EAW-2019-C002006). Five milliliters of fresh blood was collected from SPF chicks placed in anticoagulation tubes, after which the erythrocytes were washed in an equal volume of PBS. ISA brown SPF chicks were purchased from Longkol Company (Taigu, Shanxi, China). Pure erythrocytes were obtained according to the procedure described by Niu et al. [19]. The purity of erythrocytes was determined as described by Khan et al. [20]. The erythrocytes obtained were equally divided into five groups; the first group was a control group containing 50 μL of erythrocytes and 100 μL of PBS, the second group was H9N2 group containing 50 μL of erythrocytes and 100 μL of H9N2 virus, and the third, fourth, and fifth groups were supplemented with AvBD11 at final concentrations of 5, 10, and 15 μg/mL, respectively, based on the second group. DMEM was added to each group, and the final volume of each group was 1050 μL. After 2, 6, and 10 h, the red blood cells were centrifuged and washed for RNA extraction.

### 2.4. Quantitative PCR Analysis

Erythrocytes were washed three times with ice-cold PBS, and total RNA was extracted using TAKARA’s RNAiso Plus Total RNA Extraction Kit (TAKARA, Dalian, China), the RNA concentration was measured using spectrophotometry, and the RNA quality was determined based on the absorbance ratio from 260 nm to 280 nm. Reverse transcription was performed using the PrimeScript™ RT Reverse Transcription Kit (TAKARA, Dalian, China). The relative mRNA expression levels of selected genes were determined using quantitative real-time polymerase chain reaction (qPCR). Table 1 lists the primers for both the target genes and the reference genes. The relative expression level of each target gene was calculated by the 2^−ΔΔCt^ method. △Ct = Ct (target gene) – Ct (β-actin), △△Ct = △Ct (test group) − △Ct (control group).

### 2.5. Experimental Animals

A total of seventy-five 15-day-old ISA brown SPF chicks from the same batch were randomly assigned to five groups (15 chicks per group) and reared under specific pathogen-free conditions. The experiment lasted for 14 days. All experiments in this study were approved by the Institutional Animal Care and Use Committee of Experimental Animal Management at the Shanxi Agricultural University, China (Approval No SXAU-EAW-2019-C002006). These experiments were conducted according to the ARRIVE (Animal Research: Reporting of in vivo Experiments) guidelines. Group 1 was the control group (injected PBS); group 2 was the H9N2 group; groups 3, 4, and 5 were injected intramuscularly with different doses of AvBD11 (2, 4, and 8 mg/kg) according to the body weight of the chickens every day during the 3 days prior to infection with the H9N2 virus, referred to as the low-, medium-, and high-dose groups. Groups 1 and 2 were injected daily with the same dose of PBS in the same way during these three days. The route of infection and dose were the same for each test group, and 200 μL of the virus dilution was dropped into the nasal cavity of each chick. The same dose of PBS was dropped into the control group. Four chicks were randomly selected on days 3, 7, and 14 after infection for wing vein blood sampling and erythrocyte isolation.

### 2.6. Statistical Analysis

Statistical analysis was performed using SPSS 22.0 software. Duncan’s multiple comparison and one-way analysis of variance (ANOVA) were used for significance testing. The different letters above the bar graph indicate significant differences between groups. *p* < 0.05 was used as the standard for significant differences.

## 3. Results

### 3.1. Recombinant Construction Plasmid and Protein Expression

**Figure 1:**
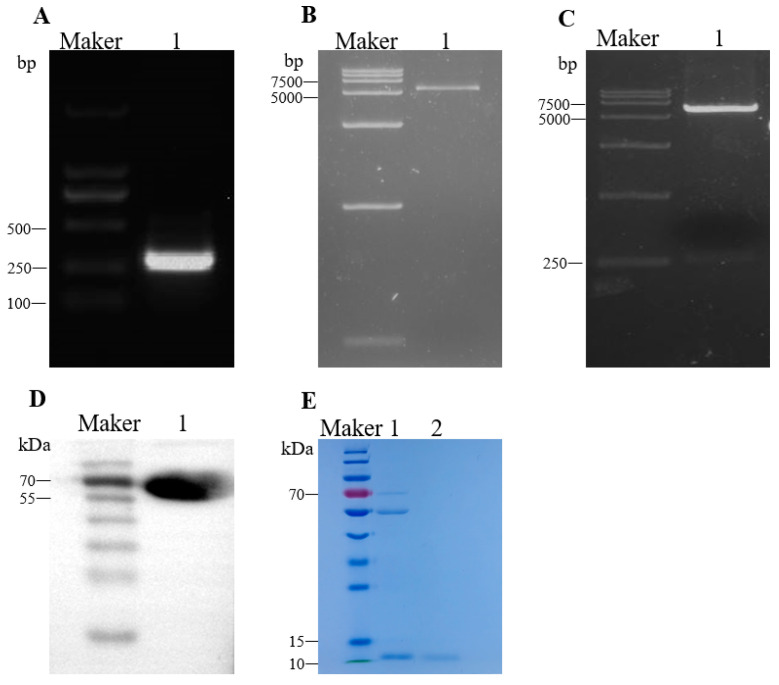
Construction of plasmid pCold-AvBD11 expressing AvBD11 fusion protein and induction of recombinant AvBD11 expression. (**A**) PCR amplification of the AvBD11 fragment; 1 lane: 246 bp. (**B**) The pCold-TF plasmid was digested to obtain a linear plasmid; 1 lane: 5769 bp. (**C**) The recombinant plasmid pCold-AvBD11 was digested with two enzymes; 1 lane: The two fragments after enzymatic cleavage. (**D**) 1 lane: identification of the recombinant protein pCold-AvBD11 by Western blot analysis. (**E**) 1 lane: enzymatic digestion of recombinant proteins; 2 lane: AvBD11 protein obtained after purification.

Mature Gga-AvBD11 is a cationic peptide composed of 82 amino acids, containing 12 cysteine residues and 6 disulfide bonds (LPRDTSRCVGYHGYCIRSKVCPFAAFGTCSWRQTCCCVDTTSDFHTCKDGGHCVSPKKIRCLEEQLGLCPLKRWTCCKEI) [11]. Firstly, we amplified the AvBD11 gene fragment (Figure 1A, lane 1); pCold-TF was double-digested in order to obtain a linear vector (Figure 1B, lane 1). We recombined the AvBD11 fusion gene fragment with the pCold linear vector and transferred it to *E. coli DH5α*. After extraction of the plasmid, it was identified by double digestion with *XhoI* and *EcoRI*, and two linear fragments of the expected size were obtained (Figure 1C, lane 1). Following the induction of protein expression in recombinant *E. coli DE3*, the bacteria underwent ultrasound treatment, and recombinant AvBD11 (approximately 70 kDa) matching the expected target protein size was identified through Western blotting (Figure 1D). Subsequently, AvBD11 protein (approximately 11 kDa) was isolated using HRV3C protease (Figure 1E, lane 2).

### 3.2. Effect of AvBD11 on the Expression of Several Cytokines in H9N2-Infected Chicken Erythrocytes

**Figure 2:**
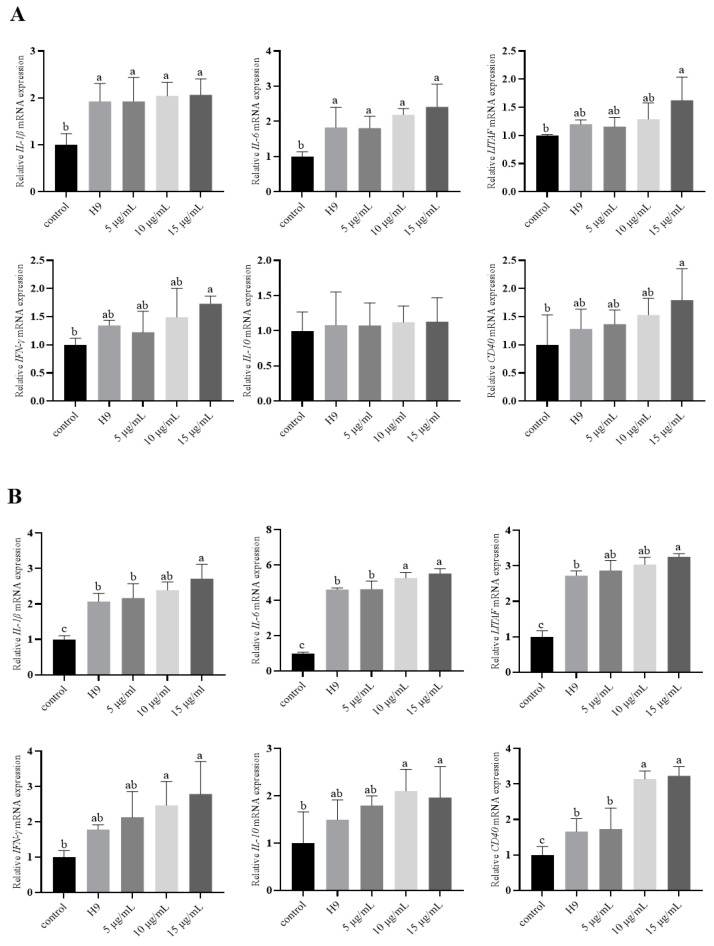

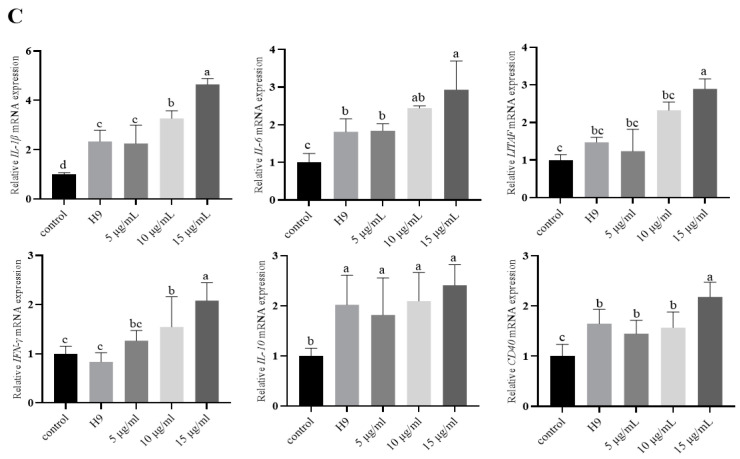
Effect of AvBD11 recombinant protein on the expression of various cytokines in H9N2-infected chicken erythrocytes. Cytokine expression was determined by qPCR. n = 4. All culture conditions were tested in triplicate. Data are expressed as mean ± SEM and represent three independent experiments. The different letters above the bar graph indicate significant differences between groups (*p* < 0.05). (**A**–**C**) Relative cytokine expression in chicken erythrocytes after 2, 6, and 10 h.

The relative expression of different cytokines varied with the time of analysis (Figure 2). At 2 h, IL-1β and Interleukin-6 (IL-6) were more significantly increased in all test groups than in the control group (p < 0.05). The addition of AvBD11 at a final concentration of 15 μg/mL resulted in significantly increased LITAF, IFN-γ, and CD40 compared to the control groups (*p* < 0.05). At 6 h, IL-1β, IL-6, LITAF, and CD40 were more significantly increased in all experimental groups than in the control group (*p* < 0.05). IL-1β, IL-6, and CD40 were more significantly increased in the 15 μg/mL AvBD11 group than in the H9 and 5 μg/mL groups (*p* < 0.05), and IL-6 was significantly more increased in the 10 μg/mL AvBD11 group than in the H9 and 5 μg/mL groups (*p* < 0.05); At 10 h, IL-1β, IL-6, IL-10, and CD40 were more significantly increased in all test groups than in the control group (*p* < 0.05). IL-1β and IFN-γ were more significantly increased in the 15 μg/mL and 10 μg/mL groups than in the H9 group. IL-1β, LITAF, IFN-γ, and CD40 were more significantly increased in the 15 μg/mL group than in the 10 μg/mL group (*p* < 0.05).

### 3.3. Effect of AvBD11 on the Expression of Various Cytokines in Erythrocytes from H9N2-Infected Chickens In Vivo

**Figure 3:**
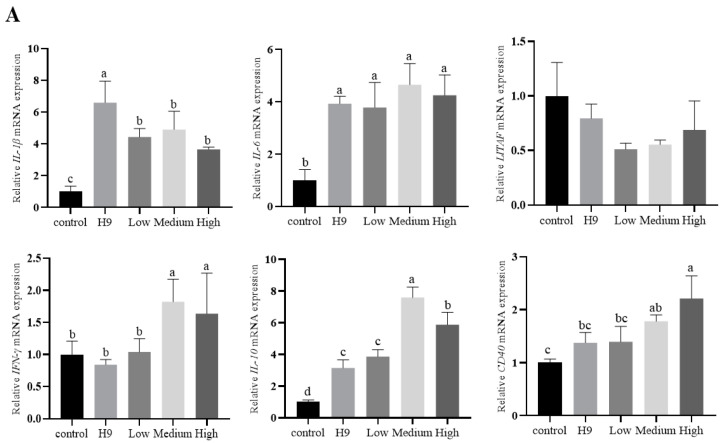

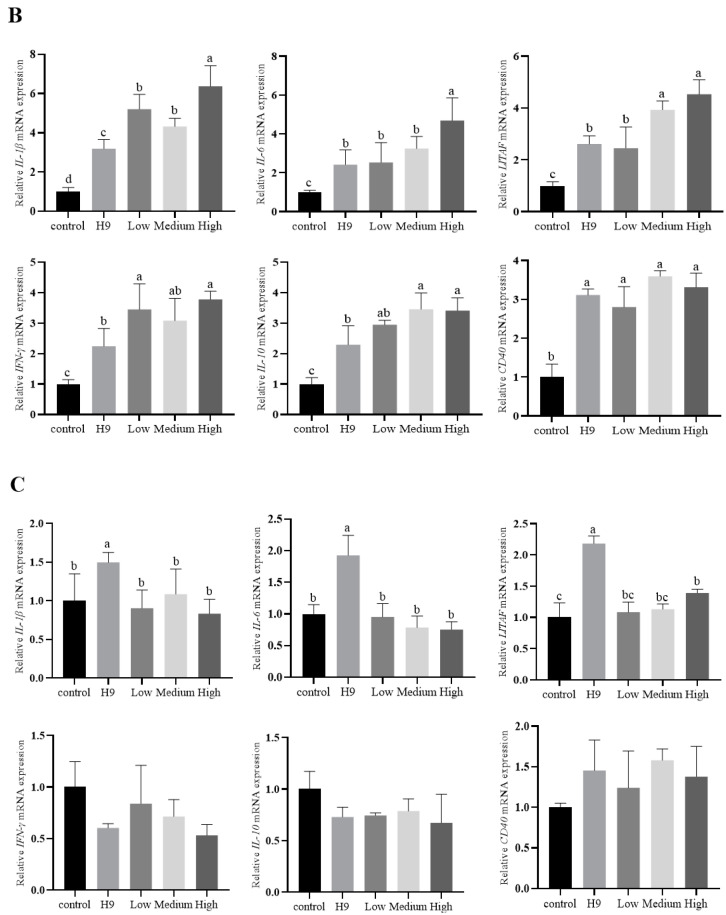
Effect of AvBD11 recombinant protein on the expression of various cytokines in erythrocytes from H9N2-infected chickens in vivo. Cytokine expression was determined by qPCR. n = 4. All culture conditions were tested in triplicate. Data are expressed as mean ± SEM and represent three independent experiments. The different letters above the bar graph indicate significant differences between groups (*p* < 0.05). (**A**–**C**) Relative cytokine expressions of chicken erythrocytes at 3, 7, and 14 days.

Three days after H9N2 infection (Figure 3A), *IL-1β*, *IL-6*, and *IL-10* were more significantly increased in all test groups than in the control group (*p* < 0.05). *IL-1β* was more significantly increased in the H9 group than in the AvBD11-treated group (*p* < 0.05). *IFN-γ* and *IL-10* were more significantly increased in the mid- and high-dose groups than in the H9 and low-dose groups (*p* < 0.05). After 7 days of infection (Figure 3B), cytokine expression levels were more significantly increased in all test groups than in the control group (*p* < 0.05). *IL-1β*, *IL-6*, *LITAF*, *IFN-γ*, and *IL-10* were more significantly increased in the high-dose group than in the H9 group (*p* < 0.05). IL-1β, *LITAF*, and *IL-10* were more significantly increased in the mid-dose group than in the H9 group (*p* < 0.05). After 14 days of infection (Figure 3C), *IL-1β*, *IL-6*, and *LITAF* were significantly increased in the H9 group compared with the other groups (*p* < 0.05).

## 4. Discussion

When the virus enters the host, the innate immune system is first activated by pattern recognition receptors. Cytokines produced at the site of infection recruit innate immune cells and antigen-presenting cells, which then transmit antigenic signals to adaptive immune cells, triggering specific humoral and cellular immune responses [21]. Cytokines play pivotal roles in immune regulation, disease defense, and tissue repair. Understanding how the chicken immune system responds to H9N2 is paramount to effectively controlling virus transmission and developing vaccines. The presence of the AvBD11 protein is predicted from the vast majority of avian genomes [22]. There are many reports on β-defensins as an assay for positive correlation with cytokine secretion produced by host cells after stimulation by bacteria or other PAMPs [23].

In this study, we investigated the effect of AvBD11 on the relative expression of cytokines (*IL-1β*, *IL-6*, *IL-10*, *LITAF*, *IFN-γ*, and *CD40*) in H9N2-infected chicken erythrocytes, both in vivo and in vitro. IL-1β is a potent pro-inflammatory cytokine that plays a critical role in the host’s defense response to infection and injury [24]. IL-6 is produced rapidly and transiently in response to infection and tissue injury and promotes host defense by stimulating the acute phase response, hematopoiesis, and immune response [25]. LITAF is a key transcriptional regulator of tumor necrosis factor-alpha (TNF-α) and can promote its transcription and expression. TNF-α is a key component of normal immune response, which can activate and regulate the immune system. However, improper or excessive production of TNF-α may cause harm, and it is known to trigger various inflammatory molecules, including other cytokines and chemokines, functionally [26]. IFN-γ is a pleiotropic cytokine with antiviral, antitumor, and immunomodulatory functions. Thus, it plays an important role in the coordination of innate and adaptive immune responses [27]. In inflammatory environments, IFN-γ triggers the activation of immune responses and stimulates the elimination of pathogens; it also prevents immune overactivation and tissue damage [28]. The main function of IL-10 is to inhibit the production and release of Th1-type immune responses and inflammatory cytokines and to exert negative immunomodulation. IL-10 targets include lymphocytes, monocytes, macrophages, mast cells, neutrophils, and eosinophils as well as releasing inflammatory mediators and Th1 immune responses. Meanwhile, IL-10 promotes antigen presentation and has an immunostimulatory effect. IL-10 acts as an anti-inflammatory factor and has important immunomodulatory effects on the body [29]. In an in vitro assay, the mRNA levels of several cytokines were significantly upregulated in chicken erythrocytes individually infected with H9N2 at different times of infection. The expression levels of *IL-1β*, *IL-6*, *LITAF*, *IFN-γ*, *IL-10* and *CD40* mRNA were significantly upregulated by the addition of H9N2 and high-dose AvBD11 treatment at 6 h. The relative expressions of *IL-1β*, *IL-6*, *LITAF*, *IFN-γ*, and *CD40* were significantly increased at 10 h. This suggests that the addition of 15 μg/mL AvBD11 promotes cytokine expression during H9N2 infection. The increase in cytokines in the short term can improve the progression of the innate immune response. On the third day of infection, *IL-1β* was more significantly increased in the H9N2-infected group than in the other test groups in the in vivo test. In contrast, both *IFN-γ* and *IL-10* were more significantly increased in erythrocytes from chickens treated with the medium and high doses than in the H9N2-infected group; after 7 days of infection, *IL-1β*, *LITAF*, and *IL-10* were more significantly increased in the medium- and high-dose groups than in the untreated and H9N2-infected groups; after 14 days of infection, the expression of *IL-1β*, *IL-6*, and *LITAF* was more significantly increased in the H9N2-infected group than in the other test groups. During the longer period of LPAI infection, AvBD11 itself or immune cells in the chicken may have cleared some of the pathogen, resulting in lower levels of pro-inflammatory cytokine expression during this period, consistent with the process of innate immune response occurring in the organism. IL-6 has been shown to have potent bio-adjuvant activity that is an enhancer of the body’s immune function [30]. It has been reported that IL-6 can effectively ameliorate lung injury caused by influenza virus infection [31], and it is hypothesized that the mRNA expression level of IL-6 is elevated in the pre-infection period, which, on the one hand, ameliorates the damage to the organism caused by viral infection and, on the other hand, strengthens the immune function of the organism and plays a regulatory role in maintaining the immune homeostasis of the organism. IFN-γ can orchestrate multiple protective functions to enhance immune responses in infection and cancer. It can exert its immunomodulatory effects by improving antigen processing and presentation, increasing leukocyte trafficking, inducing an antiviral state, enhancing antimicrobial function, and influencing cell proliferation and apoptosis [32]. AvBD11 can activate a large amount of cytokine expression in a short period of time, enabling the body to mount a rapid immune response. IL-1β acts as an amplifier of the immune response, and IL-1 is widely recognized as effective initiator of the innate immune response and the development of adaptive immune responses to address acute inflammation [33]. However, excessive IL-1β production can lead to autoimmune diseases and cause autoinflammatory disorders [34]. In in vivo tests, the AvBD11-treated groups had significantly lower *IL-1β* than the H9N2-infected group after 14 days. How do chicken AvBDs exert their immune functions? Hong et al. [35] showed that avian AvBD8 modulates immune responses in chicken macrophages by upregulating pro-inflammatory cytokines (*IL-1β*, *IFN-γ*, and *IL-12p40*) and chemokines (*CCL4*, *CXCL13*, and *CCL20*). Additionally, Western blotting and immunocytochemistry confirmed that AvBD8 activates the MAPK signaling pathway through ERK1/2 and p38. Yang et al. [36] discovered that AvBD13 activates NF-κB, induces the inflammatory cytokines IL-12 and IFN-α, and upregulates co-stimulatory molecules such as CD80 and monocyte proliferation. These effects were significantly inhibited by anti-TLR4 antibodies. In the presence of AvBD13, TLR4 expression was rapidly downregulated. AvBD13 can directly regulate monocytes and acts as an endogenous ligand for TLR4, upregulating co-stimulatory molecules and promoting monocyte proliferation. Similar to AvBD8 and AvBD13, AvBD11 is also a chicken-derived defensin. The binding of AvBD11 can influence cytokine changes in red blood cells infected with the H9N2 subtype of avian influenza virus, suggesting that AvBD11 may play an important role as an immunomodulatory factor.

## 5. Conclusions

The immune response of chicken AvBD11 to the H9N2 avian influenza virus appears to be closely associated with alterations in erythrocyte cytokine production. These cytokines play a crucial role in resisting viral infections and preserving the stability of immune system. Understanding the dynamics of this immune response could provide valuable insights into developing better strategies for combating avian influenza in chicken. To enhance poultry health and reduce the economic impact of viral infections, further investigation into the mechanisms behind these cytokine alterations may identify new targets for enhancing immune resilience.

## Figures and Tables

**Table 1:** The information on the primers used in quantitative PCR.

Gene	Primer Sequence (5′–3′)	GenBank Accession Number
*LITAF*	F:CTGAGGCATTTGGAAGCAGC	NM_204267.2
	R:GACAGGGTAGGGGTGAGGAT	
*IL-10*	F:AGGAGCAAAGCCATCAAGCA	NM_001004414.4
	R:ACCGAACGTTAAGCTGCCAT	
*IL-6*	F:GCTTGGTTAACCCTGGCTCT	NM_204628.2
	R:AAAGTGCAGAGTGTCCGACC	
*IL-1β*	F:GCCTGCAGAAGAAGCCTCG	XM_015297469.3
	R:GTGACGGGCTCAAAAACCTC	
*CD40*	F:TCCAAAACTGAGCCATGCCA	NM_204665.3
	R:GTGTGCACCAGGCAGTAGAT	
*IFN-γ*	F:CCACACCTTCCTCCAAGACA	NM_205149.2
	R:GCCTGTGAGGTTGTGGATGT	
*β-actin*	F:ATTGTCCACCGCAAATGCTTC	NM_205518.2
	R:AAATAAAGCCATGCCAATCTCGTC	

## Data Availability

The data presented in this study are available on request from the corresponding author.

## Abbreviations

The following abbreviations are used in this manuscript:

LITAF	Lipopolysaccharide-induced TNF factor
IL-10	Interleukin-10
IL-6	Interleukin-6
IL-1β	Interleukin-1β
IFN-γ	Interferon-γ
